# PD35 - In vitro effects of atorvastatin on function, proliferation and cytokine production of human peripheral blood mononuclear cells

**DOI:** 10.1186/2045-7022-4-S1-P35

**Published:** 2014-02-28

**Authors:** Vladimir Turuntas, Miodrag Colic, Sasa Vasilijic

**Affiliations:** 1 University Hospital Foca, Foca, Bosnia and Herzegovina; 2 Institute for Medical Research, Military Medical Academy, Belgrade, Serbia

## 

Statins, which are used as cholesterol-lowering agents, have anti-inflammatory and immune-regulating properties. Little comprehensive analysis has been made to investigate the impact of statins on human T lymphocytes function, their proliferation and production of Th1 and Th2 cytokines. We also investigated if the immunossupresive effects of statins are due to their impact on human T lymphocytes or proffesional antigen-presenting dendritic cells (DC) alone, or both.

Human peripheral blood mononuclear cells (MNC) proliferation was induced with phytohemagglutinin. Immature DC were cultivated from monocytes of healthy donors. DC maturation was induced by lipopolysaccharide (LPS; 1 μg/mL). Unstimulated and LPS-stimulated DC were treated with atorvastatin (1-10 μM). Ability to induce T cell proliferation and the secretion of cytokines were performed in mixed leukocyte reaction.

Atorvastatin reduced phytohemagglutinin-stimulated MNC proliferation, dose dependently. The results showed that atorvastatin, only in high concentrations, strongly inhibit allogenic lymphocytes proliferation irrespectively which stimulators are used: immature or mature dendritic cells. Low concentrations of atorvastatin even stimulated proliferation when the immature dendritic cells were used as stimulators. Atorvastatin have influence on DC differentiation and maturation by changing expression and mean fluorescence intensity of costimulatory, adhesive and maturation molecules such as HLA-DR, CD80, CD83, CD86, CD40, CD54 and CD25. Atorvastatin affects allostimulatory activity of both immature and mature MoDC but in completely oposite way, using different DC/Ly ratio, and change production of TNF-α by immature DC and IL-18 by mature DC and not affect IL-10 and IL-12 production. Atorvastatin decreased the level of IFN-γ, increase the level of IL-10, and not affected the level of IL-4 in DC culture supernatants when the atorvastatin was added during maturation. If the atorvastatin was added in monocite culture, no significant change in cytokine level was observed.

Our data provide strong evidence that atorvastatin can act as an immunomodulator by reducing, but in low dosage stimulating T lymphocite proliferation, inhibiting the immune response of Th1, decreasing the expression of co-stimulatory molecules, and changing DC cytokine secretion, which can help in better understanding how to take advantage of these new mechanistic insights in increased use of statins in therapeutic strategy in different immune/inflamatory disorders in future.

